# Activation of autophagy inhibits the activation of NLRP3 inflammasome and alleviates sevoflurane-induced cognitive dysfunction in elderly rats

**DOI:** 10.1186/s12868-023-00777-5

**Published:** 2023-01-28

**Authors:** Junjie Zhou, Chao Zhang, Xu Fang, Naixin Zhang, Xiaoxi Zhang, Zhaoqiong Zhu

**Affiliations:** 1 Affiliated Hospital of Zunyi Medical University, 149 Dalian Road, Huichuan District, Zunyi, 563000 Guizhou China; 2 Zunyi Medical University, 6 Xuefu West Road, Xinpu New District, Zunyi, 563000 Guizhou China

**Keywords:** Autophagy, Elderly rat, Nucleotide‐binding oligomerization domain‐like receptor protein 3 inflammasome, Sevoflurane

## Abstract

**Aims/introduction:**

As a common complication in elderly patients after surgery/anesthesia, postoperative cognitive dysfunction (POCD) is mainly characterized by memory, attention, motor, and intellectual retardation. Neuroinflammation is one of the most uncontroversial views in POCD. The sevoflurane-induced neurotoxicity has attracted widespread attention in recent years. However, its mechanism has not been determined. This study aimed to observe the effects of sevoflurane on cognitive function and the changes in inflammatory indices and autophagy protein expression in the prefrontal cortex in aged rats.

**Method:**

Before the experiment, D-galactose was diluted with normal saline into a liquid with a concentration of 125 mg/kg and injected subcutaneously into the neck and back of rats for 42 days to establish the aging rat model. Morris water maze experiments were performed, including positioning navigation (5 days) and space exploration (1 day). The POCD model was established by 3.2% sevoflurane inhalation. The rats were treated with or without MCC950, a potent and selective nucleotide‐binding oligomerization domain‐like receptor protein 3 (NLRP3) inhibitor, followed by autophagy agonists and autophagy inhibitors. The expression levels of inflammasome-related protein NLRP3 and autophagy-related proteins LC3B and P62 were detected to test the behavior of rats with a water maze.

**Results:**

We found that sevoflurane exposure affected learning and working memory ability in aged rats. We also observed microglia activation in the prefrontal cortex. NLRP3 protein expression was significantly upregulated after sevoflurane inhalation. NLRP3 inflammasome activation induced increased expression and mRNA expression of cleaved Caspase-1 and inflammatory cytokines IL-1β and IL-18, and increased secretion of peripheral proinflammatory cytokines. The inhibitor MCC950 was used to improve cognitive ability and inflammation in rats and inhibit the secretion of cytokines. In addition, we demonstrated that significant inhibition of autophagy (decreased LC3-II/I and increased P62) was accompanied by increased activation of NLRP3 inflammasomes and more severe neural cell damage. However, autophagy inhibitor rapamycin administration to activate autophagy resulted in the inhibition of NLRP3 inflammasomes, ultimately attenuating neuronal injury.

**Conclusions:**

The activation of autophagy suppressed the formation of NLRP3 inflammasomes. It also alleviated cognitive impairment in aged rats.

**Supplementary Information:**

The online version contains supplementary material available at 10.1186/s12868-023-00777-5.

## Introduction

Postoperative cognitive dysfunction (POCD) is a common neurological complication after anesthesia and surgery, more common in the elderly. It is characterized by deterioration in memory, attention, and information processing speed [1]. These age-related postoperative complications affect the health care system and socioeconomic development with the rapid growth of the aging population worldwide [2]. More studies have shown that neuroinflammation is one of the pathophysiological mechanisms of cognitive dysfunction in elderly rodents after anesthesia and surgery [3, 4]. However, how neuroinflammation attacks the elderly brain after anesthesia and surgery remains to be clarified. Microglia are the main resident immune cells in the central nervous system, which are closely related to the initiation of neuroinflammation in the brain after exposure to many exogenous stimuli [5, 6]. In addition, the imbalance of neuroinflammatory response derived from microglia is involved in the cognitive dysfunction in several age-related neurodegenerative diseases [7–9].

Sevoflurane is widely adopted in clinics because of its less stimulation and fast anesthesia induction. However, many studies have shown that sevoflurane is one of the causes of POCD after anesthesia/surgery. Pattern recognition receptor (PRR) is the hub of the whole innate immune response. Glial cells can express a variety of PRRs, such as nucleotide‐binding oligomerization domain‐like receptor (NLR) [9]. The nucleotide‐binding oligomerization domain‐like receptor protein 3 (NLRP3) of the NLR family containing the pyrin domain is the most representative [10]. The NLRP3 inflammatory body is a cytoplasmic complex of an early inflammatory response, which contains NLRP3, ASC, and Caspase-1 [11]. During activation, NLRP3 forms a complex with its adapter ASC. When the body is abnormally stimulated, NLRP3 inflammatory bodies trigger the cleavage and activation of Caspase-1, make IL-1β and IL-18 mature, and trigger inflammatory response [12, 13]. NLRP3 inflammatory bodies induced inflammatory diseases of the central nervous system in studying the prefrontal cortex of rats with traumatic brain injury [14]. As a consequence, exploring the role of NLRP3 inflammatory bodies in the prefrontal cortex in sevoflurane-induced cognitive dysfunction in rats was worth exploring.

Autophagy is an intracellular degradation event that plays a housekeeping role in eliminating protein aggregates and abnormal organelles and is an important cytoprotective pathway [15]. Enhanced mitophagy benefits learning and memory in rodents, and its dysfunction disrupts neuronal homeostasis and predisposes individuals to neurodegenerative or neuropsychiatric diseases [16]. Therefore, elucidating the sevoflurane-induced NLRP3 inflammasome signaling pathway in the rat prefrontal cortex may provide a new therapeutic target for treating POCD.

In recent years, D-galactose (D-gal)-induced simulated aging rat model has been widely used in the study of age-related diseases, indicating that chronic D-gal exposure can induce premature aging in rats, similar to natural aging rats [17]. With the increase in POCD incidence in elderly patients, an easily accessible animal model for the study of cognitive dysfunction in the elderly is urgently needed.

Based on the aforementioned discussion, it was hypothesized that sevoflurane initiated the NLRP3/Caspase-1 inflammatory pathway through mitochondrial autophagy to cause cognitive dysfunction in the aging brain after sevoflurane exposure.

## Materials and methods

### Animals, experimental design, and experimental grouping

Four-month-old male Sprague–Dawley rats were purchased from Changsha Tianqin Biotechnology Co., Ltd. Besides, all the experiments were approved by the Experimental Animal Committee of Zunyi Medical University and were in accordance with the guidelines for the nursing and use of experimental animals of the National Research Committee. Four rats in each cage were randomly fed under a 12-h light/dark cycle (23–25 °C) to provide enough food and water. In addition, the rats were randomly divided into groups and experiments in an unbiased, double-blind manner.

D-galactose (Solarbio, D8310, Beijing, China) was diluted with normal saline into a liquid with a concentration of 125 mg/kg and continuously injected subcutaneously into the neck and back of rats for 42 days to establish an aging rat model. The rats in the experimental group were placed in a thermostatically sealed anesthetic box after starving for 12 h, which was connected to an anesthetic machine (Drager, Julian, Germany) and an exhaust absorption device. The rats were induced with 3:1 pure oxygen and air mixed with 6% sevoflurane (Lunambett Pharmaceuticals Co., Ltd, Shandong, China) and maintained with 3:1 pure oxygen and air mixed with 3.2% sevoflurane for 6 h. The anesthetic gas monitor (Drager Medical GmbH, Canada) was continuously monitored during anesthesia to prevent the retention of CO_2_ and deep anesthesia. The rats in the control group were treated with a 3:1 pure oxygen and air mixture for 6 h. The rats in the positive control group were intraperitoneally injected with MCC950 (MCE, HY-12815A, NJ, USA) for 30 min and then inhaled with 3:1 pure oxygen and air for 6 h. The rats in the treatment group were treated with pure oxygen and air at 3:1 and 3.2% sevoflurane for 6 h, 30 min after the intraperitoneal injection of MCC950; the fixed concentration of MCC950 was 10 mg/kg.

The rats were randomly divided into 4 groups in experiment 1, with 12 rats in each group: (1) Ctrl, blank group; (2) MCC950, MCC950 negative control group; (3) Sev, sevoflurane group; and (4) MCC950 + Sev, MCC950 pretreatment group. In experiment 2, the rats were divided into 4 groups, with 12 rats in each group: (1) Ctrl, blank group; (2) 3-MA, 3 methyladenine group; (3) Sev, sevoflurane group; and (4) Rapa, rapamycin group.

### Morris water maze test

The Morris water maze (MWM) device comprised a round black water tank (120 cm in diameter and 60 cm in depth). In addition, the water maze was divided into four quadrants, one of which was selected to place a 15-cm-diameter escape platform 1 cm below the surface of the water. The rats in each group continued to carry out the positioning navigation experiment for 5 days, 24 h after D-gal injection. The specific operation was that the rats were put in the water facing the pelvic wall from a fixed point in each quadrant. The time it took for each rat to reach the escape platform was observed and recorded. The rats were guided to the platform for 30 s if they did not find the platform within 120 s. In addition, the experiment in the next quadrant was carried on.

The spatial exploration test was carried out 24 h after the intervention of sevoflurane. The specific operation was to withdraw the platform from the original quadrant. In addition, the rats entered the water from any quadrant facing the pelvic wall. The percentage and times of rats crossing the original platform quadrant within 120 s and the total distance and speed of swimming in the water were observed and recorded.

The specific operation of the working memory test was to put the platform into the diagonal quadrant of the original platform quadrant and keep the rats down in any quadrant. In addition, the operators observed and recorded the escape platform time again after the rats escaped the platform rest for 30 s; the second time was recorded as the escape latency time.

The aforementioned test results were recorded using a video tracking system (Taimeng Technology Co., Ltd., Chengdu, China).

### Methods of tissue sampling and euthanasia in rats

After the end of behavioral study, 100 mg/kg sodium pentobarbital was injected into the abdomen of the rats for deep anesthesia. After the rats were anesthetized, the rats were quickly fixed on the operating table for laparotomy and abdominal aorta blood was taken to prepare for the experimental dosage. After that, the rats were continued to bleed to death, and then the prefrontal cortex of the rats was removed by rapid decapitation, the tissue was separated after 30 min or within 24 h for follow-up experiments.

### Nissl staining

The prefrontal cortex of rats was fixed with 4% paraformaldehyde, dehydrated, and embedded in paraffin for sections. Besides, the slices were dewaxed in xylene, hydrated with gradient ethanol, washed with pure water, and dripped with Nissl staining solution (Solarbio, G1436) to evaluate the neuronal damage of the prefrontal cortex. Finally, Nissl staining was observed under a microscope (Olympus Corporation, BX63, Tokyo, Japan).

### Immunohistochemical staining

The prefrontal cortex of the rat brain was fixed with 4% paraformaldehyde, embedded in paraffin, cut into 4- to 5-μm-thick sections, dewaxed with xylene, and dehydrated with gradient alcohol. The sections were incubated with Iba-1 antibody (1:100, Abcam, ab178846, Cambridge, UK) overnight in the refrigerator at 4 ℃ and then incubated with biotin-labeled secondary antibody (Abcam, ab205718) for 40 min. Moreover, the operators conducted diaminobenzidine (DAB, ZLI-9017, Beijing, China) staining, hematoxylin re-staining, and sealing of tablets.

### Enzyme-linked immunosorbent assay (ELISA)

The concentrations of tumor necrosis factor (TNF)-α and interleukin (IL)-6 in the supernatant and serum of the prefrontal lobe of bran were detected using an ELISA kit (Jianglaibio Co., Ltd., CK-E31063; CK-E30219, Shanghai, China).

### Real-time reverse transcription–polymerase chain reaction

The total RNA of the rat prefrontal cortex was extracted using an RNAiso Plus Trizol RNA extraction kit (TaKaRa Bio, Japan). The PrimeScript RT kit was used to reverse transcribe cDNA. Besides, an SYBR Green polymerase chain reaction (PCR) kit (Sangon Biotech, Shanghai, China) was used for real-time fluorescence quantitative reverse transcription PCR in the detection system (Bio-Rad, USA). The primer sequences (Sangon Biotech) are listed in Table 1.

**Table 1:** List of primer sequences used for RT-PCR

Accession number	Gene name	Primer sequence (F: forward; R: reverse)
NM_001191642.1	*NLRP3*	F: TGT CAG GAT CTC GCA R: AGT GAA GTA AGG CCG GAA T
NM_012762.3	*Caspase-1*	F: GAC AAG ATC CTG AGG GCA AA R: GGT CTC GTG CCT TTT CCA TA
NM_031512.2	*IL-1β*	F: CTG GAC TCG TGG GAT GAT G R: GGG ATT TTG TCG TTG CTT GT
NM_019165.1	*IL-18*	F: AAC GAA TCC CAG ACC AGA C R: AGA GGG TAG ACA TCC TTC CAT
NM_012589.2	*IL-6*	F: CAG TTGCCT TCT TGG GGA CT R: GGT CTG TTG TGG GGT GGT ATC
NM_012675.3	*TNF-α*	F: CCA CCA CGC TCT TCT GTC R: GCT ACG GGC TTG TCA CTC
NM_031144.3	*β-actin*	F: TGT CAC CAA CTG GGA CGA TA R: GGG GTG TTG AAG GTC TCA AA

### Western blot (WB)

First of all, the WB experiment involved in this paper adopted the trimmed membrane for western blotting, which is a routine experimental method in our laboratory. Second, blots were cut off before hybridization with antibodies in the paper involved protein stripe images, we have provided the original blots. The specific method is the proteins were quantified with BCA kits (Solarbio, PC0020), separated by polyacrylamide gel electrophoresis (Bio-Rad), and transferred to PVDF membrane (Millipore, USA) after cleavage of the rat prefrontal cortex with a mixture of protease inhibitors (RIPA:PMSF = 100:1). Then, 5% skimmed milk powder was used for sealing after electroporation, followed by incubation with antibodies against NLRP3 (Novus, NBP2-12446, CO, USA), Caspase-1 (Novus, NB100-56564, Cambridge, UK), IL-1β (BIOSS, bs-20449R, Beijing, China), IL-18 (Proteintech, 10663-1-AP, Wuhan, China), IL-6 (BIOSS, bs-0782R), TNF-α (Abcam, ab178846, Cambridge, UK), β-actin (Proteintech, 20,536–1-APa), GAPDH (Proteintech, 10494-1-AP), LC3B (HuaBio, ET1701-65, Hangzhou, China), and SQSTM1/p62 (HuaBio, R1309-8).

### Separation of mitochondria

Mitochondria isolation from the prefrontal cortex: Intact cortical mitochondria were isolated from fresh brain tissue using a tissue mitochondrial isolation kit. In brief, 50 mg of prefrontal cortex tissue was homogenized in 2 mL of pre-ice-cold protein solution. The homogenate was centrifuged at 6000*g* for 5 min at 4 °C. The collected supernatant containing cytosolic proteins was then destroyed by adding 1.5 mL of pre-ice-cold 1.5 mL of protein solution to the supernatant to resuspend and destroy the particles. After further centrifugation at 11,000*g* for 10 min at 4 °C, the supernatant was collected and centrifuged at 6000*g* for 10 min. The pellet (containing mitochondria) was resuspended in 750 μL of mitochondrial purification buffer and centrifuged at 14,000*g* for 15 min. A pellet or band containing mitochondria was formed in the lower part of the tube and transferred to a new tube. The suspension was washed three times with 1.5 mL of mitochondrial storage buffer by centrifugation at 8000*g* for 10 min. Highly purified mitochondria were resuspended in mitochondrial storage buffer and stored at − 80 °C until further use.

### Transmission electron microscopy (TEM)

After the anesthetized rats were fixed on the operating table, the brain tissue was quickly removed, and the prefrontal cortex of the rats was stripped. The trimmed tissue was placed in a 3% glutaraldehyde fixation solution, fixed at room temperature for 2 days, and then placed at 4 °C for later use. The steps were repeated twice: 70% acetone for 15 min → 80% acetone for 15 min → 90% acetone for 15 min → 100% acetone for 1 min → embedding with epoxy resin embedding agent → dodecane succinate hardening agent → diethyl amphetamine accelerator → butyldiester phthalate plasticizer → 45 °C drying for 12 h → 60 °C drying for 36 h → slicing into 40-nm sections → mesh supporting film → dropping dye solution (uranyl acetate + lead nitrate) for 15 min → observing under a transmission electron microscope (Hitachi Limited, Japan).

### Statistical analyses

The experimental data were analyzed and processed using SPSS18.0 statistical software. In addition, the measurement data were expressed as mean ± standard deviation. The repeated measurements of the water maze were performed using analysis of variance after the spherical test. The pairwise comparison between groups was analyzed using the least significant difference test. The difference was statistically significant when *P* < 0.05.

## Results

### Effects of sevoflurane on cognitive function in aged rats

**Fig. 1:**
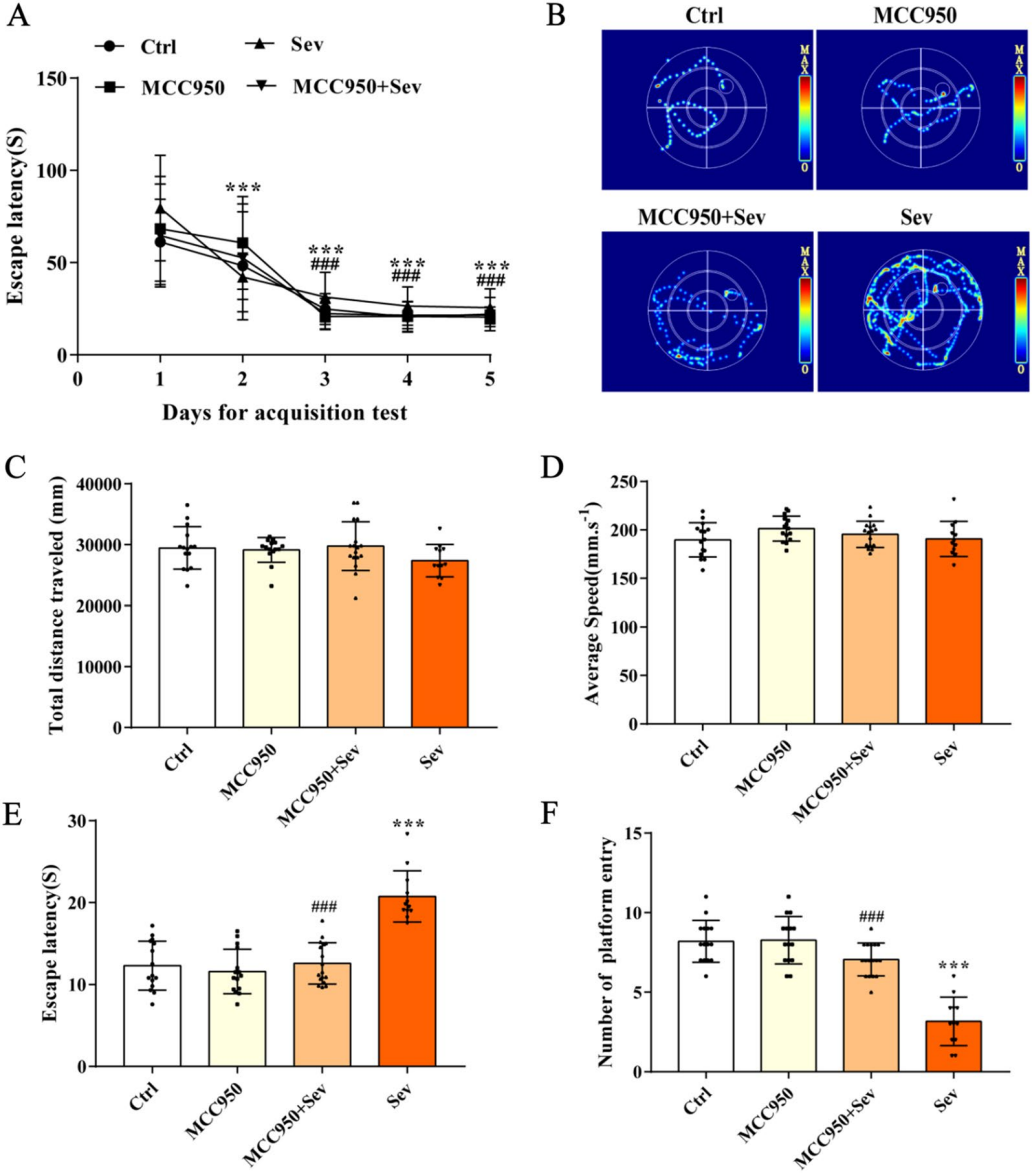
Sevoflurane can lead to cognitive changes in rats, but not without changing motor performance. **A** Positioning navigation test results. **B** Movement trajectory of water maze of rats in working memory. **C** Total swimming distance of rats in each group in the water maze test. **D** Average swimming speed of rats in each group in the water maze test. **E** Escape latency of rats in each group in the water maze test. **F** Number of times traversing the original platform. Data are expressed as mean ± SD. *P* < 0.05 indicated a statistically significant difference. ***Compared with day 1, ^###^compared with day 2; ***compared with Ctrl group, and ^###^compared with Sev group

The results of the MWM test are shown in Fig. 1B. The escape latency of rats in each group decreased with the extension of learning time in the positioning navigation test for five consecutive days before sevoflurane intervention (*P* < 0.05). As shown in Fig. 1E and F, the escape latency increased (*P* < 0.05) and the time needed to escape the platform in working memory increased (*P* < 0.05) in the Sev group compared with the Ctrl group. As shown in Fig. 1C and D, no significant difference was found in swimming speed and percentage of entering the original platform quadrant among the three groups in space exploration (*P* > 0.05). The escape latency decreased (*P* < 0.05), the frequency of crossing the original platform increased (*P* < 0.05), and the time needed to escape the platform in working memory decreased (*P* < 0.05) in the MCC950 + Sev group compared with Sev group. These results suggested that sevoflurane could reduce the learning and memory ability of rats, but did not change the movement and speed of rats.

### Effects of sevoflurane on nerve injury and microglial activation in the prefrontal cortex of aged rats

**Fig. 2:**
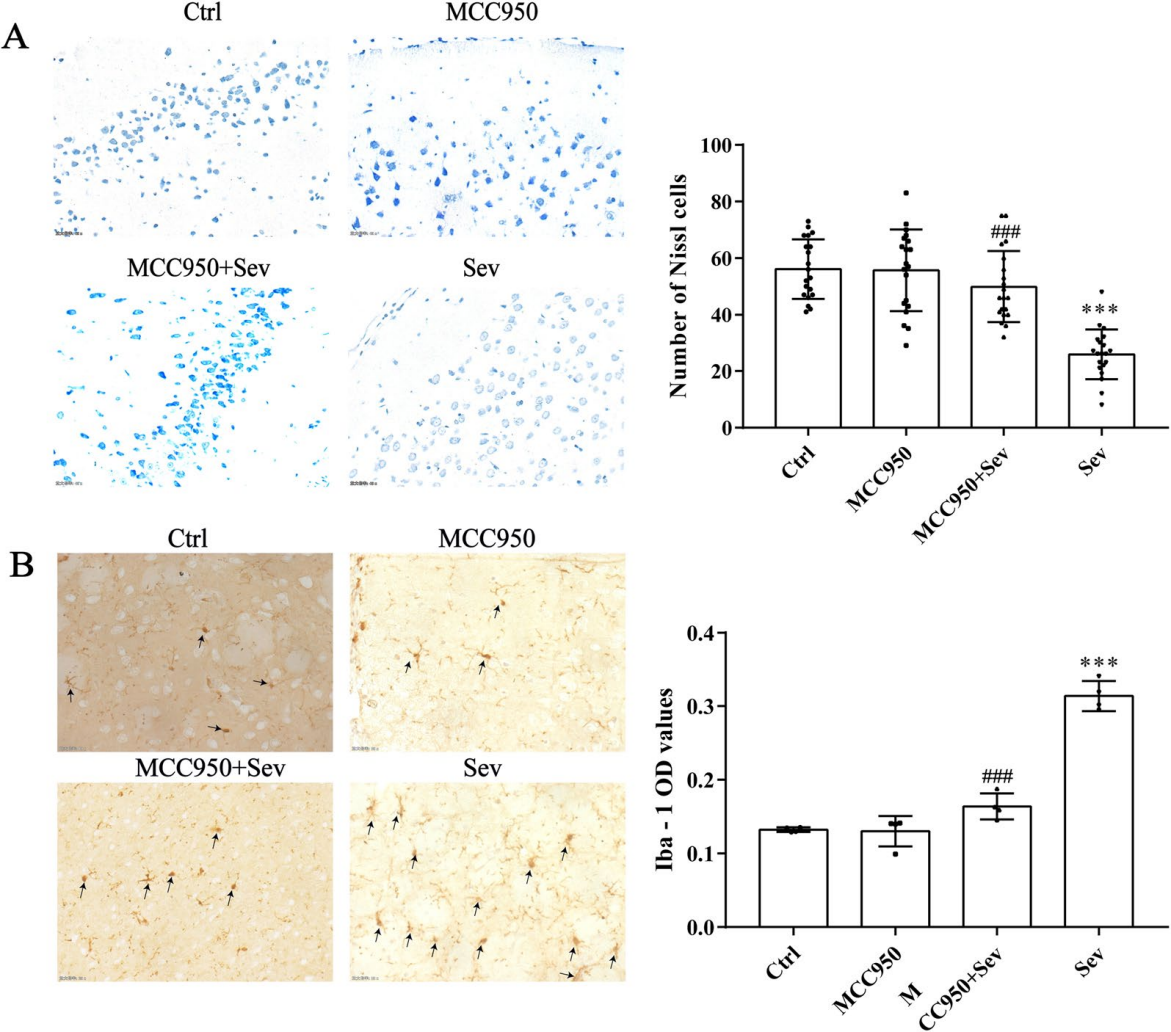
Sevoflurane causes neuronal dysfunction in the prefrontal cortex of rats. **A** Nissal staining results in the prefrontal cortex of rats in each group (× 400). **B** Immunohistochemical images of Iba-1-positive cells in the prefrontal cortex of rats in each group (× 400). Data are expressed as mean ± SD; *n* = 4 for each experimental group. ***Compared with the Ctrl group, ^###^compared with the Sev group. *P* < 0.05 indicated a statistically significant difference

Nissl staining was used to evaluate the damage to prefrontal cortex neurons in rats. The results of Nissl staining are shown in Fig. 2A. The number of Nissl bodies in the prefrontal cortex was significantly lower in the Sev group compared with the Ctrl group (*P* < 0.05). The number of Nissl bodies in the prefrontal cortex increased in the MCC950 + Sev group (*P* < 0.05) compared with the Sev group. It was suggested that MCC950 could reverse the sevoflurane-induced damage to cerebral neurons in rats. In addition, Iba-1 is a specific marker of microglia activation, and its positive rate can be adopted to evaluate the degree of microglial activity. Compared with the Ctrl group, amoeba-like microglia were seen, and the optical density (OD) value significantly increased (*P* < 0.05). The immunohistochemical results are shown in Fig. 2B. The activation degree of microglia and the OD value decreased in the MCC950 + Sev group (*P* < 0.05) compared with the Sev group. It was suggested that MCC950 could inhibit the overactivation of microglia.

### MCC950 inhibited the release of proinflammatory cytokines induced by sevoflurane and the activation of inflammatory mediators

The levels of proinflammatory cytokines in the brain tissue and serum were measured by Western blot and PCR to evaluate the inhibitory effect of MCC950 on the activation of microglia in the sevoflurane-induced rat prefrontal cortex. The results clearly showed that sevoflurane could significantly increase the protein and mRNA levels of IL-6 and TNF-α (*P* < 0.05) and increase inflammation (*P* < compared with Ctrl group. In addition, the inflammatory levels in the brain tissue and serum decreased significantly after MCC950 intervention (*P* < 0.05) compared with the Sev group, indicating that MCC950 could significantly reduce the levels of central and peripheral inflammatory factors.

**Fig. 3:**
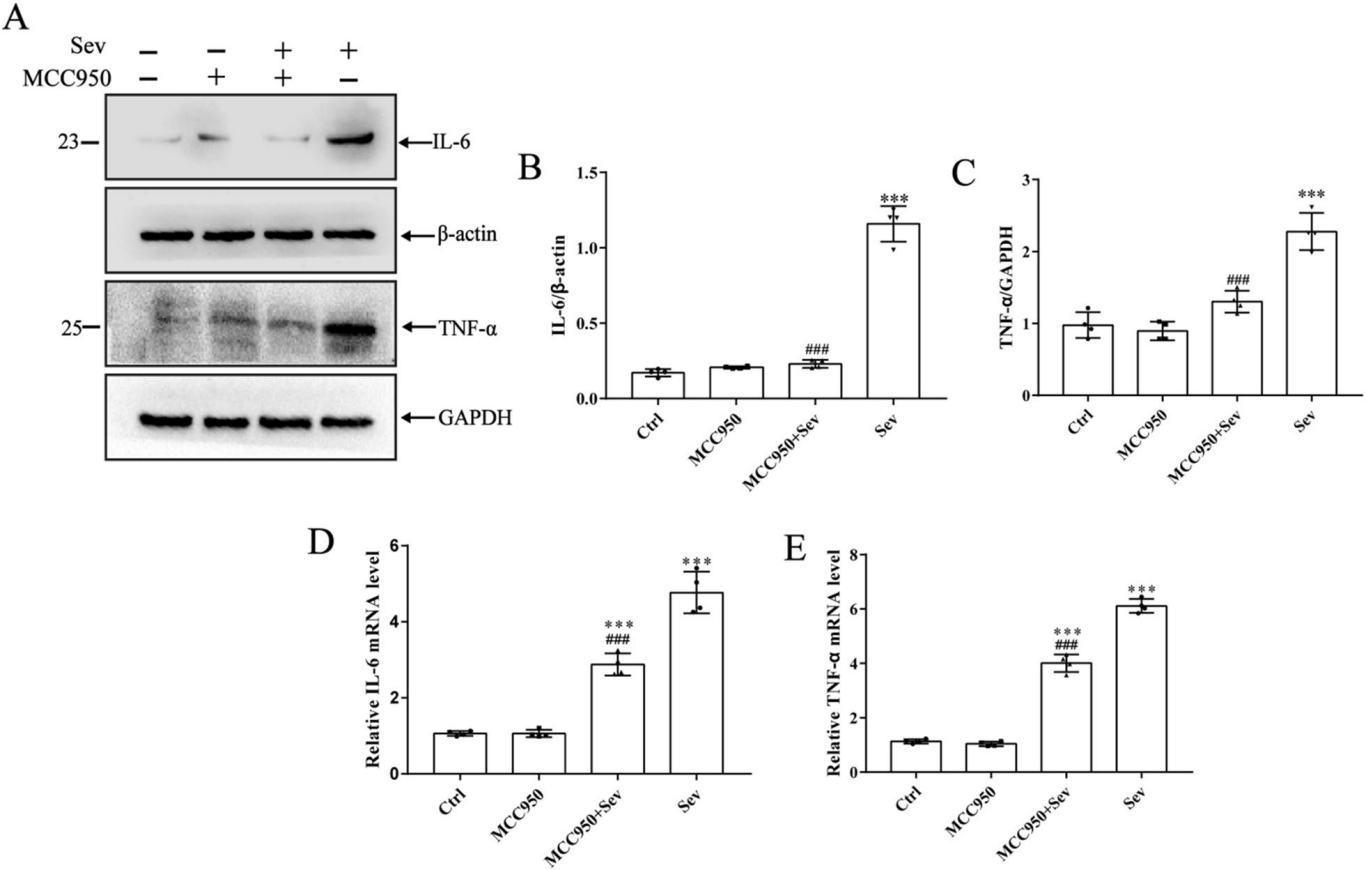
Sevoflurane can cause brain damage in the prefrontal cortex in rats. Brain tissues from prefrontal cortex were homogenized, and inflammatory proteins levels were detected by immunoblotting and PCR. **A**–**C** Proinflammatory cytokines were expressed in four groups of rats. Western blot images were cropped to remove irrelevant sections of the image and display only the proteins of interest. Loading control: protein extracted from prefrontal cortex tissue from sham subjects treated with saline (lane1). Tissue used for Western blot analysis was collected 24-h after the last drug-administration. **D**–**E** PCR results of proinflammatory factors. Data are expressed as mean ± SD; *n* = 4 for each experimental group. ***Compared with the Ctrl group, ^###^compared with the Sev group. *P* < 0.05 indicated a statistically significant difference

As shown in Fig. 3A and B, the levels of IL-6 and TNF-α in brain tissue were detected by Western blot and PCR to evaluate the inhibitory effect of MCC950 in the prefrontal cortex of rats. The results showed that sevoflurane significantly induced the protein and mRNA levels of IL-6 and TNF-α (*P* < 0.05) compared with the control group. In addition, the brain tissue and serum inflammation significantly reduced after MCC950 intervention (*P* < 0.05) compared with the Sev group. Similarly, the levels of inflammatory factors in the serum of rats were detected using ELISA. As shown in Table 2, sevoflurane increased the levels of serum inflammatory factors IL-6 and TNF-α (*P* < 0.05), and MCC950 significantly decreased the expression of serum inflammatory factors, indicating that sevoflurane increased the levels of central and peripheral inflammatory factors.

**Table 2:** Serum levels of inflammatory factors in the rats of each group (ng/mL, *n* = 12, mean ± SD)

Group	IL-6	TNF-α
Ctrl	69.20 ± 10.81	54.85 ± 12.45
MCC950	64.95 ± 10.78	60.11 ± 9.77
MCC950 + Sev	85.93 ± 8.51^***###^	102.73 ± 10.89^***###^
Sev	140.37 ± 13.77***	184.33 ± 26.01***

***Compared with the Ctrl group, ^###^compared with the Sev group. *P* < 0.05 indicated a statistically significant difference

### NLRP3 inflammatory bodies were an important part of the inflammatory process, which are highly expressed in inflammatory diseases

**Fig. 4:**
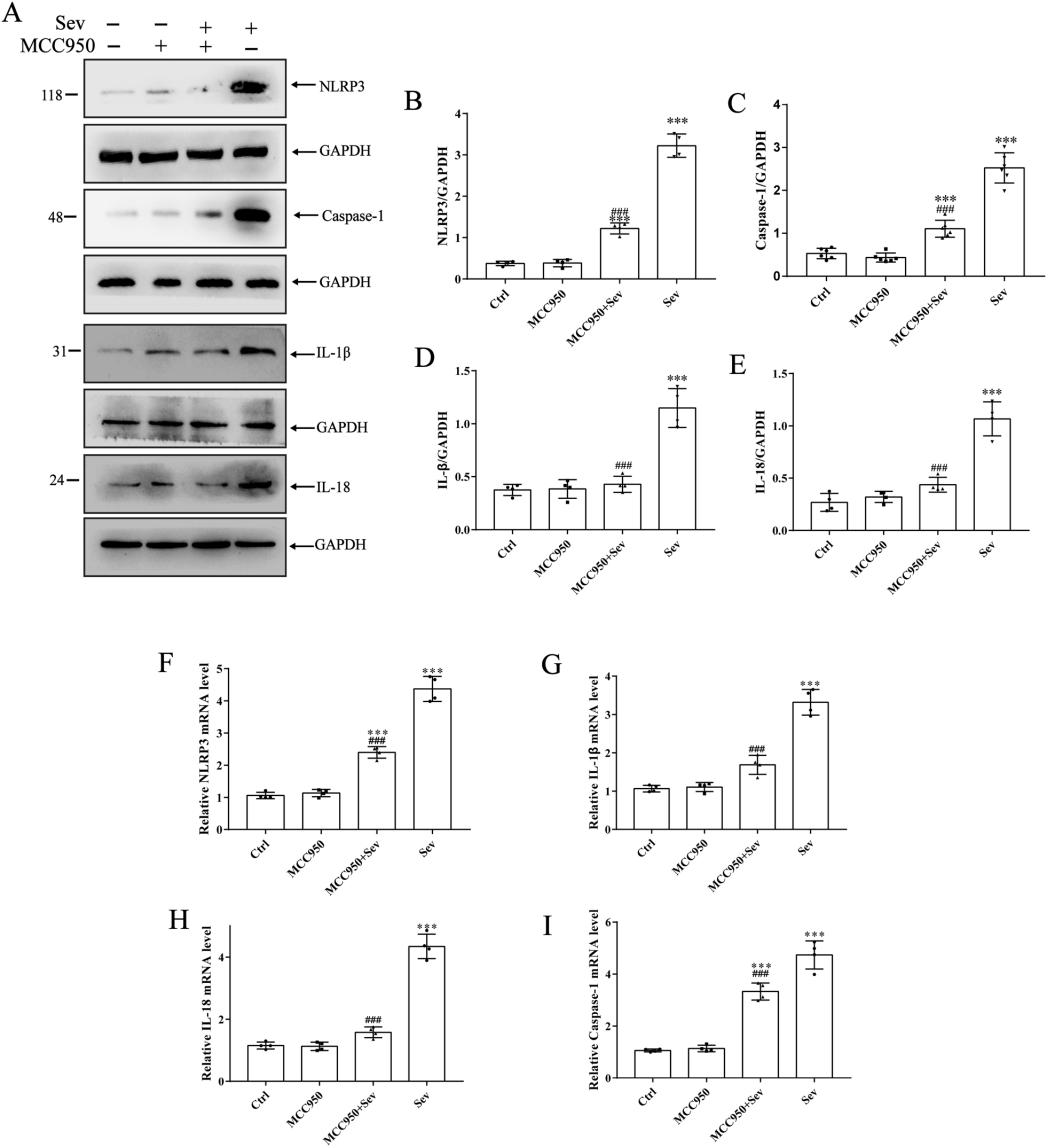
Sevoflurane can increase the protein expression of NLRP3 inflammasome and its downstream inflammatory factors in prefrontal cortex. **A**–**E** Expression levels of NLRP3 inflammatory factors, related components, and downstream cytokine proteins. Western blot images were cropped to remove irrelevant sections of the image and display only the proteins of interest. Loading control: protein extracted from prefrontal cortex tissue from sham subjects treated with saline (lane1). Tissue used for Western blot analysis was collected 24-h after the last drug-administration. **F**–**I** NLRP3 inflammatory factors, related components, and downstream cytokine PCR results. Data are expressed as mean ± SD; *n* = 4 for each experimental group. ***Compared with the Ctrl group, ^###^compared with the Sev group. *P* < 0.05 indicated a statistically significant difference

Further, whether the activation of inflammatory bodies by sevoflurane was inhibited by MCC950 was analyzed. The results as shown in Fig. 4A–E. The protein levels of NLRP3, Caspase-1, IL-1β, and IL-18 (*P* < 0.05) increased in the Sev group compared with the Ctrl group. The corresponding rising trend in PCR (Fig. 4F–I) was obtained after sevoflurane exposure (*P* < 0.05). However, it was reversed after pretreatment with MCC950, eliminating the increase in the protein and mRNA levels of NLRP3, Caspase-1, IL-1β, and IL-18 (*P* < 0.05).

### Activation of mitophagy attenuated neuronal injury by inhibiting NLRP3 inflammasome in rats

**Fig. 5:**
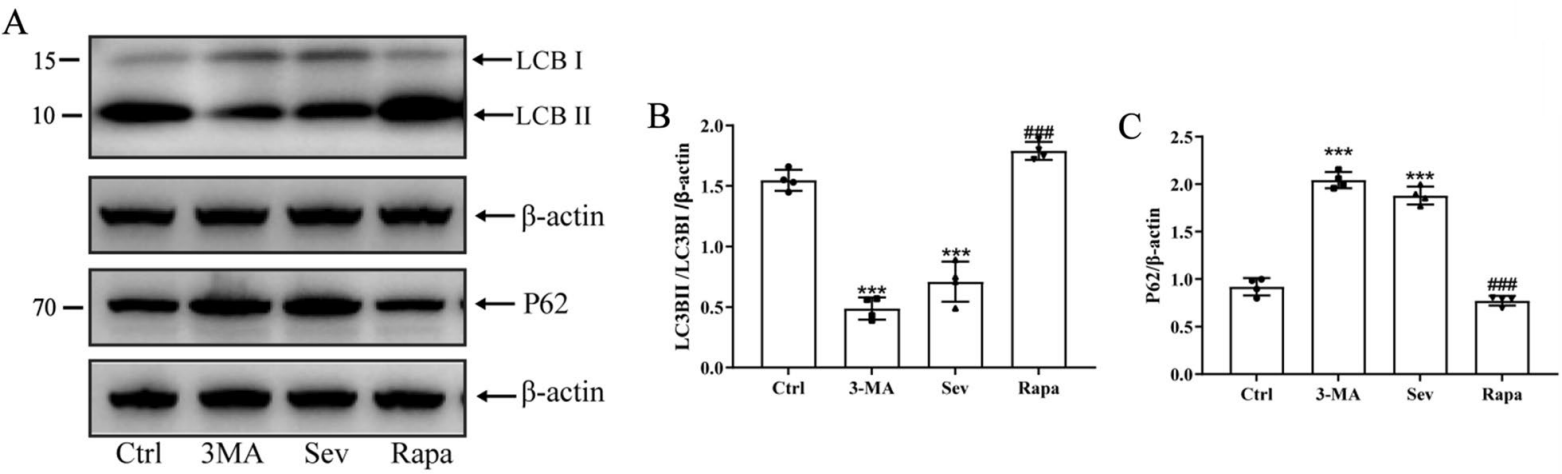
Sevoflurane can inhibit mitochondrial autophagy related proteins in prefrontal cortex of rats. **A** Representative Western blot images showing LC3B and NLRP3. LC3BI and LC3BII are LC3B subunits. The ratio of LC3BII/LC3BI represents the degree of autophagy. **B**–**C** Protein abundance statistics of LC3B and P62. Western blot images were cropped to remove irrelevant sections of the image and display only the proteins of interest. Loading control: protein extracted from prefrontal cortex tissue from sham subjects treated with saline (lane1). Tissue used for Western blot analysis was collected 24-h after the last drug-administration. These results suggested that sevoflurane reduced the degree of mitophagy in the brain. *Compared with the Ctrl group, *P* < 0.05; ^#^compared with the Sev/3-MA group, *P* < 0.05

**Fig. 6:**
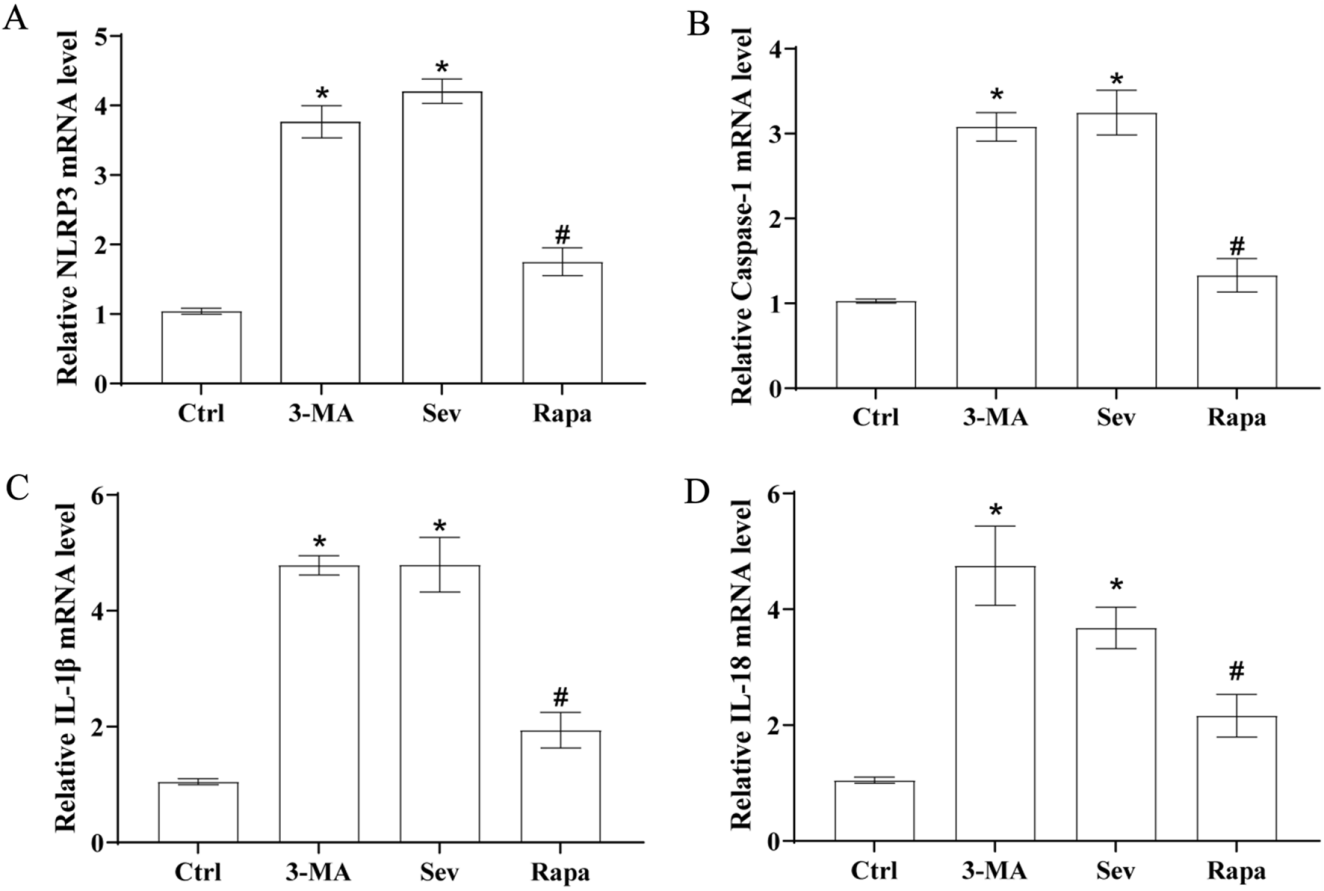
Sevoflurane can increase the mRNA expression of NLRP3 inflammasome and its downstream related proteins and inflammatory indicators in the prefrontal region of rats. **A**–**D** Transcript mRNA levels of NLRP3, Caspase-1 IL-1β, and IL-18 in the prefrontal cortex. Ctrl values are represented as blank control normalized values. The other values are fold changes relative to the blank control mean, suggesting that sevoflurane increased the mRNA expression of brain inflammation-related proteins and aggravated neuroinflammation. *Compared with the Ctrl group, *P* < 0.05; ^#^compared with the Sev/3-MA group, *P* < 0.05

**Fig. 7:**
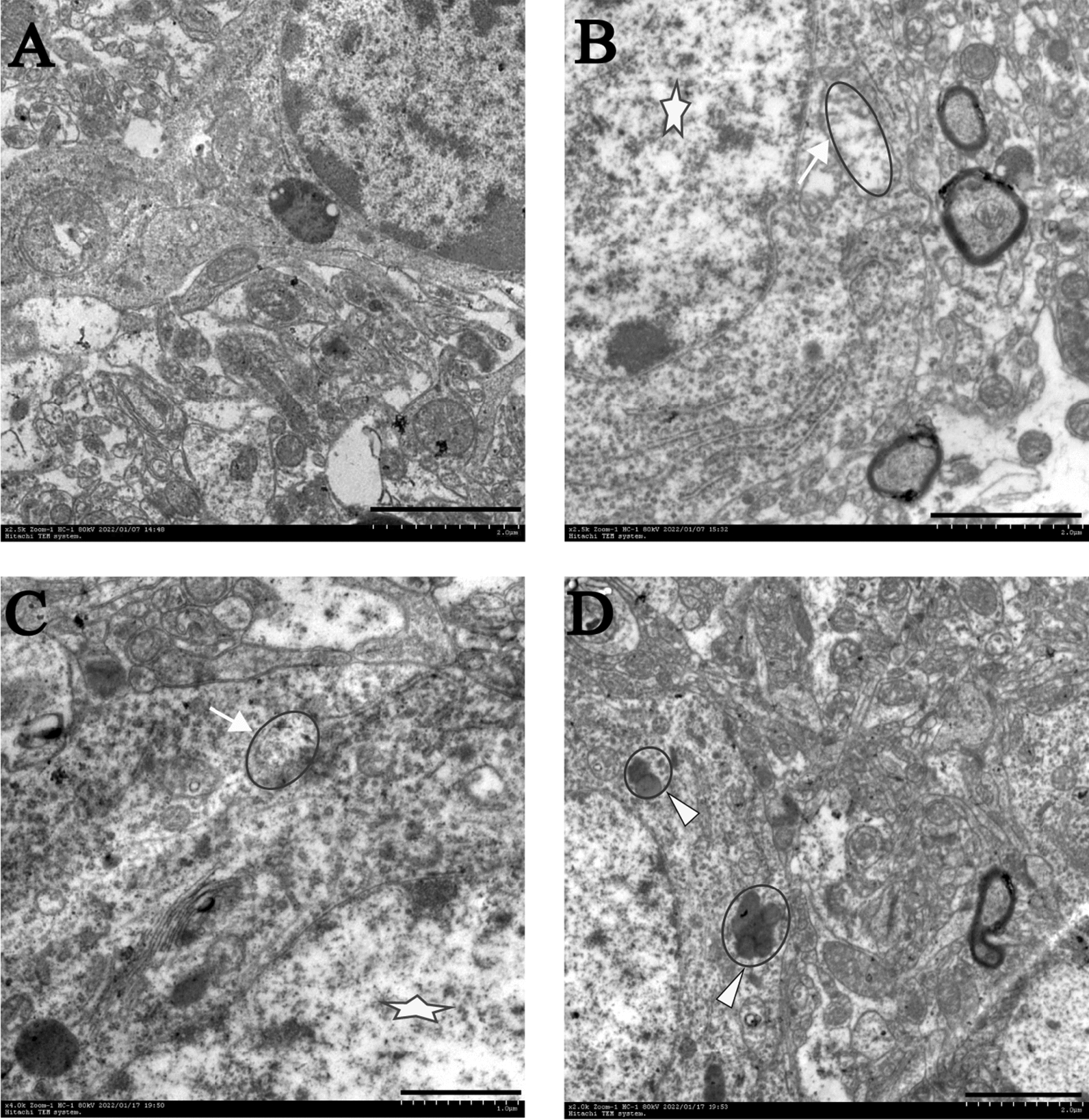
TEM images of the prefrontal cortex in rats. **A** Normal control prefrontal cortex neuron, displaying abundant organelles such as mitochondria, endoplasmic reticulum, and ribosomes. The nucleus is large and round, the density of chromatin is uniform, and nucleolus is clear. **B** Chromatin arrangement disorder and nucleolus disappearance (shown with star) and the mitochondrial crista was broken, and even vacuoles appeared (shown with arrow) is shown in the prefrontal cortex cells of 3-MA rats. **C** The mitochondria showed swelling, vacuolation and loss of crestthe mitochondrial matrix was disordered (shown with arrow) in cells of the prefrontal cortex from sevoflurane rats. **D** The neurons were basically normal, mitochondrial phagosomes and lysosomes were observed, and mitophagy increased (shown with triangle). Scale bar, 2000 nm

The rats in the sevoflurane and rapamycin groups were treated with sevoflurane for 6 h before receiving rapamycin (autophagy activator, 2 mg/kg, i.p. daily for up to 3 days) to investigate the causal relationship between mitophagy and NLRP3 inflammasome in the cortical region and its effect on POCD. The rats in the 3-MA group were treated with sevoflurane for 6 h before receiving 3-MA (autophagy inhibitor, 15 mg/kg, i.p. daily for up to 3 days). As expected, rapamycin treatment significantly enhanced the level of LC3-II/I protein and decreased the level of P62, indicating that rapamycin effectively activated autophagy (Fig. 5A–C). In addition, rapamycin treatment decreased NLRP3 inflammasomes (NLRP3, Caspase-1, as well as increased release of IL-1β and IL-18) in the cortical region of sevoflurane-treated rats, suggesting that activated autophagy mediated the inhibition of NLRP3 inflammasomes (Fig. 6A–D). Finally, transmission electron microscopy was used to observe autophagy in the prefrontal cortex. The results clearly showed that rapamycin treatment also alleviated neuronal damage and increased autophagosomes; the degree of cell damage was lower than that in the Ctrl group (Fig. 7). Given the importance of the NLRP3 inflammasomes in neuroinflammation, these observations suggested that mitophagy activation might attenuate sevoflurane-induced cognitive impairment by inhibiting the NLRP3 inflammasomes.

## Discussion

POCD refers to patients without mental disorders before the surgery, whose brain function is disordered due to various factors [18]. It can result in reversible acute mental disorder syndrome after the surgery, which is often manifested as a dysfunction of learning, memory, cognitive, and social abilities; it is common in elderly patients and is also considered the prophase of Alzheimer's disease [19]. Some anesthetics commonly adopted in the clinic, such as sevoflurane, can cause postoperative POCD in elderly patients [20]. At present, the POCD model is mainly established through sham operation, but this model can easily ignore the separate role of narcotic drugs in cognitive dysfunction. In previous studies, D-gal was used to establish the model of aging rats, and the learning and cognitive abilities of aged rats were evaluated using a water maze. The results showed that the escape latency of rats was gradually shortened and tended to be stable on the third, fourth, and fifth days, indicating that the learning and memory of rats in each group were at a unified baseline. After correlation processing, spatial exploration and working memory tests were carried out in each group. The results showed no significant difference in swimming speed and entry into the original platform quadrant among the three groups. However, the escape latency increased and the number of times crossing the original platform decreased in the Sev group compared with the Ctrl group. Consequently, the learning, memory, and cognitive abilities of these rats anesthetized with sevoflurane decreased. It was suggested that sevoflurane exposure alone successfully produced the phenotype of cognitive dysfunction. The results also showed that the escape latency decreased in the MCC950 + Sev group compared with the Sev group. However, the number of times crossing the original platform increased, suggesting that MCC950 could improve the learning, memory, and cognitive abilities of aged rats after sevoflurane anesthesia.

Microglia are the innate immune cells of the central nervous system and are extremely sensitive to pathological and physiological stimuli. They play a key role in maintaining neural homeostasis and responding to external stimuli [21]. The activation of microglia often occurs under abnormal brain stimulation. In the central nervous system, this activation process is marked by the changes in the function and morphology of microglia [22]. Moderate activation of microglia is essential for the maintenance of neuroplasticity. However, excessive microglial activation may increase the levels of inflammatory factors such as IL-6 and TNF-α [23]. In addition, the excessive production of these cytokines is a marker of cerebral nervous system diseases. As a consequence, the selective decrease in cytokine secretion can control neuroinflammatory diseases. In this experiment, microglia were observed using Iba-1, a promoter used to distinguish microglia from other nerve cells in the prefrontal cortex. In addition, the number of microglia increased significantly and showed amoeba-like changes in the Sev group compared with the Ctrl group. However, the number of microglia decreased in the MCC950 + Sev group compared with the Sev group. The results showed that MCC950 inhibited the sevoflurane-induced activation of microglia in the prefrontal cortex. Then, the protein and gene expression levels of IL-6 and TNF-α in the prefrontal cortex significantly increased, as detected using Western blot and PCR. The serum levels of IL-6 and TNF-α were detected using ELISA. At the same time, MCC950 reversed the changes in the levels of these proteins and genes and peripheral inflammation induced by sevoflurane, which partly explained the alleviating effect of MCC950 on sevoflurane-induced microglial activation.

Pyroptosis is an inflammatory form of programmed cell death, which is considered to be related to brain neuron injury and glial cell activation [24]. Glial cells express PRRs, such as NLR, which are downstream mediators of inflammatory bodies. In inflammatory body complexes, the inflammatory bodies containing NLRP3 are the most widely studied inflammatory bodies [25]. Therefore, NLRP3 inflammatory bodies are used to explore the pathogenesis of many complex diseases. MCC950 is a selective NLRP3 inflammatory body inhibitor, which inhibits the activation of NLRP3 inflammatory bodies by blocking NLRP3-induced protein oligomerization. It displayed a protective effect against ischemia–perfusion injury of lung [26], brain [27], heart [28], kidney [29], and liver [30]. MCC950 also showed an anti-inflammatory protective effect in sevoflurane-induced activation of the NLRP3/Caspase-1 pathway in this study. Most importantly, MCC950 treatment could prevent neuronal damage and cognitive dysfunction induced by sevoflurane.

Basal autophagy helps continuously deliver damaged proteins and organelles to lysosomes for degradation. In the mammalian nervous system, autophagy disorder may lead to neurodegeneration and cause extensive neuronal damage, which is essential for maintaining normal function and countering neurodegenerative homeostasis. Mammalian target of rapamycin kinase (mTOR), an autophagy regulator, receives input from different signaling pathways, particularly substances that sense the energy state of cells to trigger or stop protein synthesis [31]. MTOR kinase, a downstream target of phosphatidylinositol 3-kinase (PI3K) and Akt kinase pathways, is activated by neurotrophin and growth factor receptors to promote cell growth, differentiation, and survival, while downregulating apoptotic signaling [32, 33]. Mitophagy is generally considered to be the main mechanism of mitochondrial quality control. It is the targeted phagocytosis and destruction of mitochondria via autophagy; a similar "eat me" signal appears as the autophagy mechanism [34]. At present, 3-methyladenine (3-MA), one of the autophagy inhibitors, is used to inhibit autophagosome formation by interrupting PI3K/Akt/mTOR signaling [35, 36].

The activation of the conversion of inactive form LC3 I into lipopolysaccharide-active form LC3 II occurs in the isolated membrane in the initial stage of vesicle formation and ends with LC3 II binding to the outer and inner layers of intact autophagic double-membrane vesicles, representing the completion of autophagosome formation or other autophagy-derived autophagy [37]. In this study, the LC3B II/LC3B I ratio and SQSTM1/P62 protein level in the prefrontal cortex of rats were evaluated. The results showed that the P62 protein level significantly increased and the LC3B II/LC3B I protein ratio significantly decreased in the Sev group, which were reversed by rapamycin treatment. These results suggested that sevoflurane-induced mitochondrial autophagy activated the NLRP3 signaling pathway. Other studies reported that the autophagy-lysosomal pathway might be involved in releasing IL-1β after activating the NLRP3/Caspase-1 inflammasome pathway in chronic cerebral ischemia [38, 39]. In this study, we found that sevoflurane aggravated the mRNA overexpression of NLRP3, Caspase-1, IL-1β, and IL-18. The autophagy agonist rapamycin decreased the mRNA overexpression.

In conclusion, this study demonstrated that sevoflurane activated the NLRP3 signaling pathway by inhibiting mitophagy, increased the degree of neuronal injury in the prefrontal cortex, and exacerbated the generation of neuroinflammation in the brain. Rapamycin enhanced mitochondrial autophagy and significantly reduced neuroinflammation, suggesting that rapamycin-mediated mitophagy played a protective role in sevoflurane-induced neuroinflammation. This study further elucidated a new mechanism of mitophagy involvement in the NLRP3 signaling pathway in sevoflurane-induced cognitive dysfunction in rats (Additional file 1).

## Supplementary Information

**Additional file 1.** Western blot of the original exposure

## Data Availability

The datasets used and/or analyzed in the present study are available from the corresponding author upon reasonable request.
