# Correction: Activation of autophagy inhibits the activation of NLRP3 inflammasome and alleviates sevoflurane-induced cognitive dysfunction in elderly rats

**DOI:** 10.1186/s12868-023-00801-8

**Published:** 2023-05-22

**Authors:** Junjie Zhou, Chao Zhang, Xu Fang, Naixin Zhang, Xiaoxi Zhang, Zhaoqiong Zhu

**Affiliations:** 1 Affiliated Hospital of Zunyi Medical University, 149 Dalian Road, Huichuan District, Zunyi, 563000 Guizhou China; 2 Zunyi Medical University, 6 Xuefu West Road, Xinpu New District, Zunyi, 563000 Guizhou China

**Correction: *BMC Neurosci *24, 9 (2023**)

10.1186/s12868-023-00777-5

Following publication of the original article [1], the authors reported that the order of affiliations was inverted. The corrected affiliations are the following:

^1^Affiliated Hospital of Zunyi Medical University, 149 Dalian Road, Huichuan District, 563,000, Zunyi, Guizhou, China.

^2^Zunyi Medical University, 6 Xuefu West Road, Xinpu New District, 563,000, Zunyi, Guizhou, China

The original article [1] has been updated.
