# Supplementary material for: Activation of autophagy inhibits the activation of NLRP3 inflammasome and alleviates sevoflurane-induced cognitive dysfunction in elderly rats
Source: BMC Neurosci. 2023 Jan 28;24:9. doi: 10.1186/s12868-023-00777-5 (PMC9883890; doi:10.1186/s12868-023-00777-5)
Supplement: Supplementary file 1 — Additional file 1. Western blot of the original exposure [file 12868_2023_777_MOESM1_ESM.pdf]

Western blots original band

IL-6

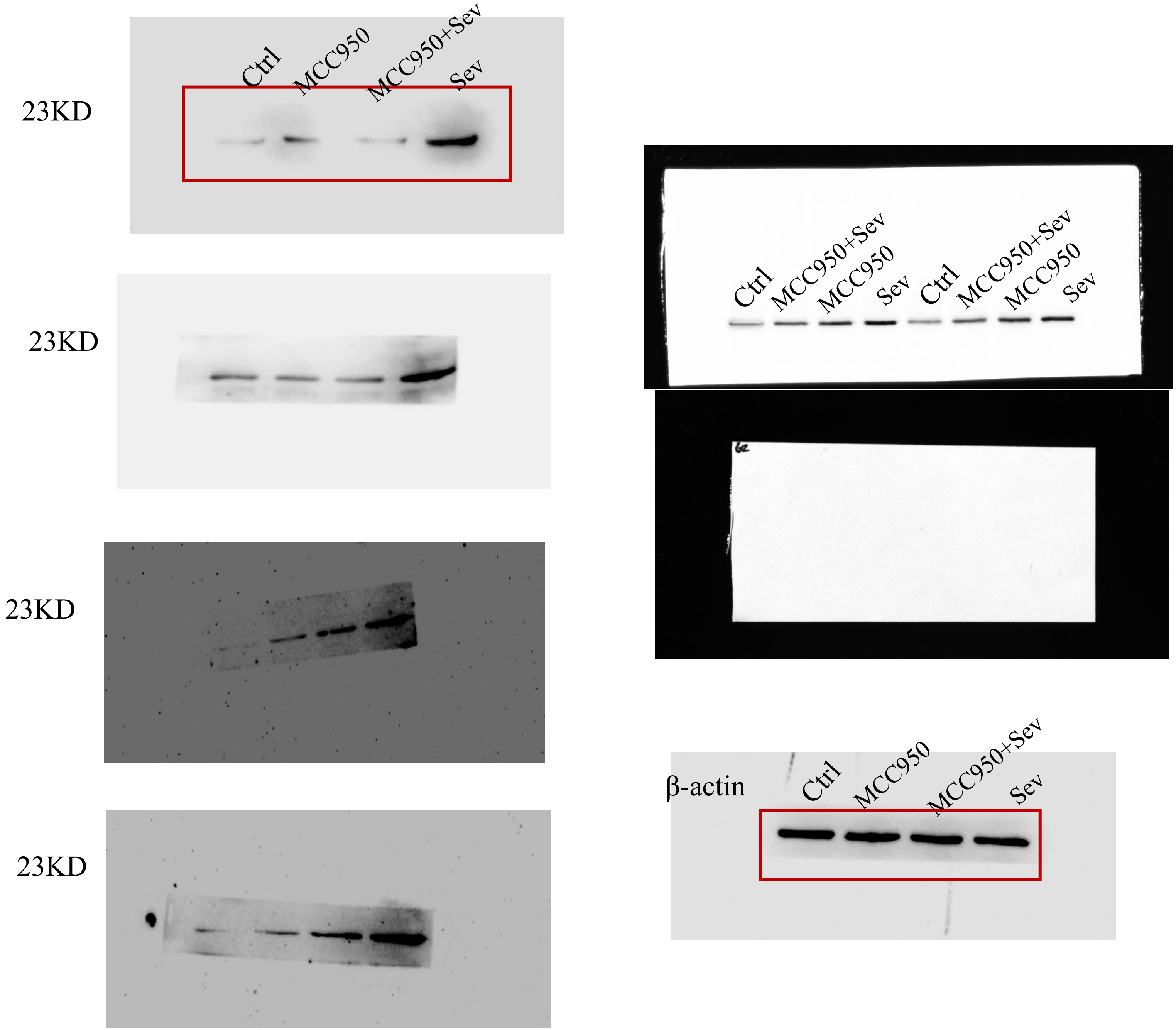

23KD
Ctrl
MCC950
MCC950+Sev
Sev
23KD
23KD
23KD
Ctrl
MCC950+Sev
MCC950
Sev
Ctrl
MCC950+Sev
MCC950
Sev
β-actin
Ctrl
MCC950
MCC950+Sev
Sev

TNF-α

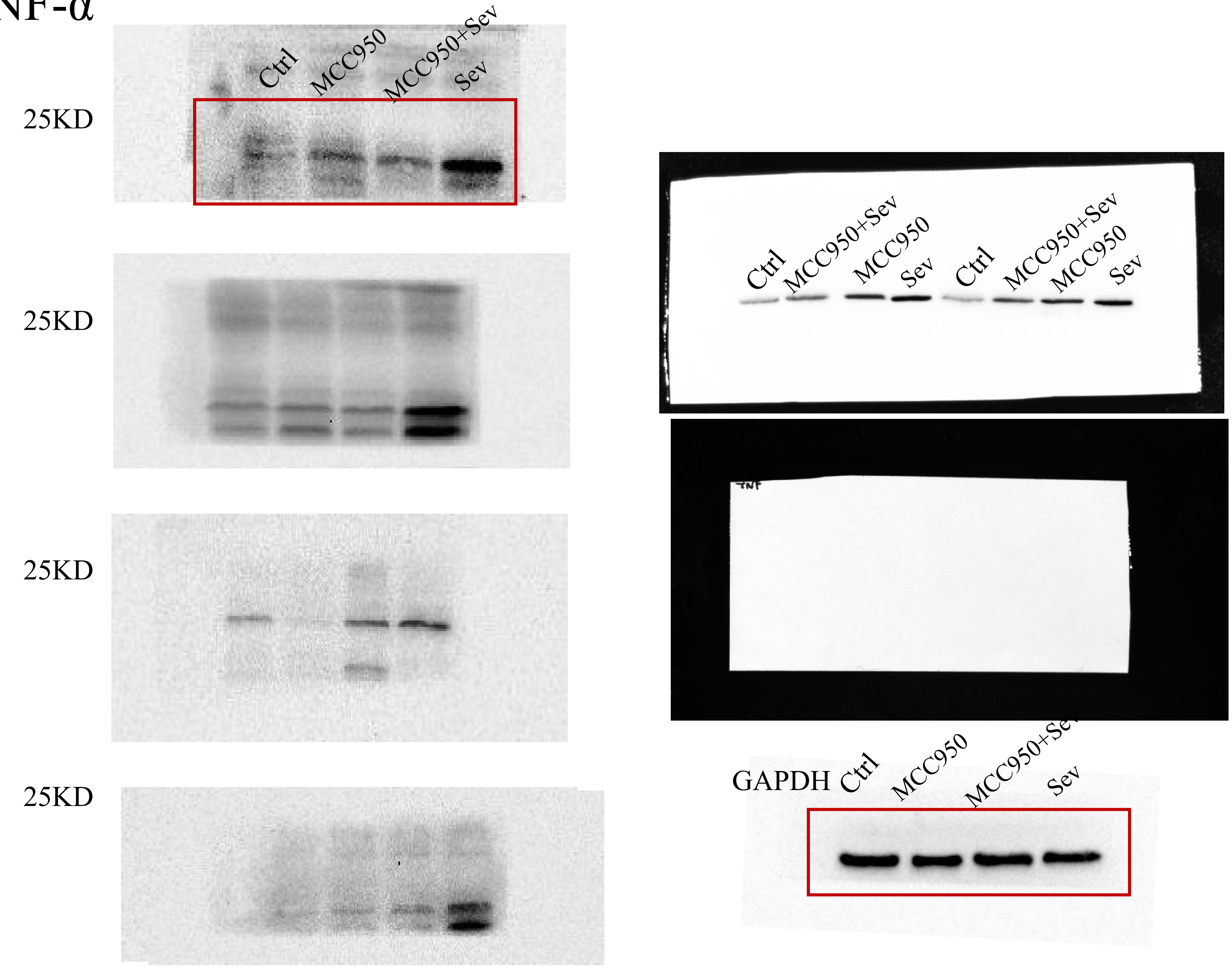
Ctrl
MCC950
MCC950+Sev
Sev
25KD
25KD
25KD
25KD
Ctrl
MCC950+Sev
MCC950
Sev
Ctrl
MCC950+Sev
MCC950
Sev
GAPDH
Ctrl
MCC950
MCC950+Sev
Sev

NLRP3

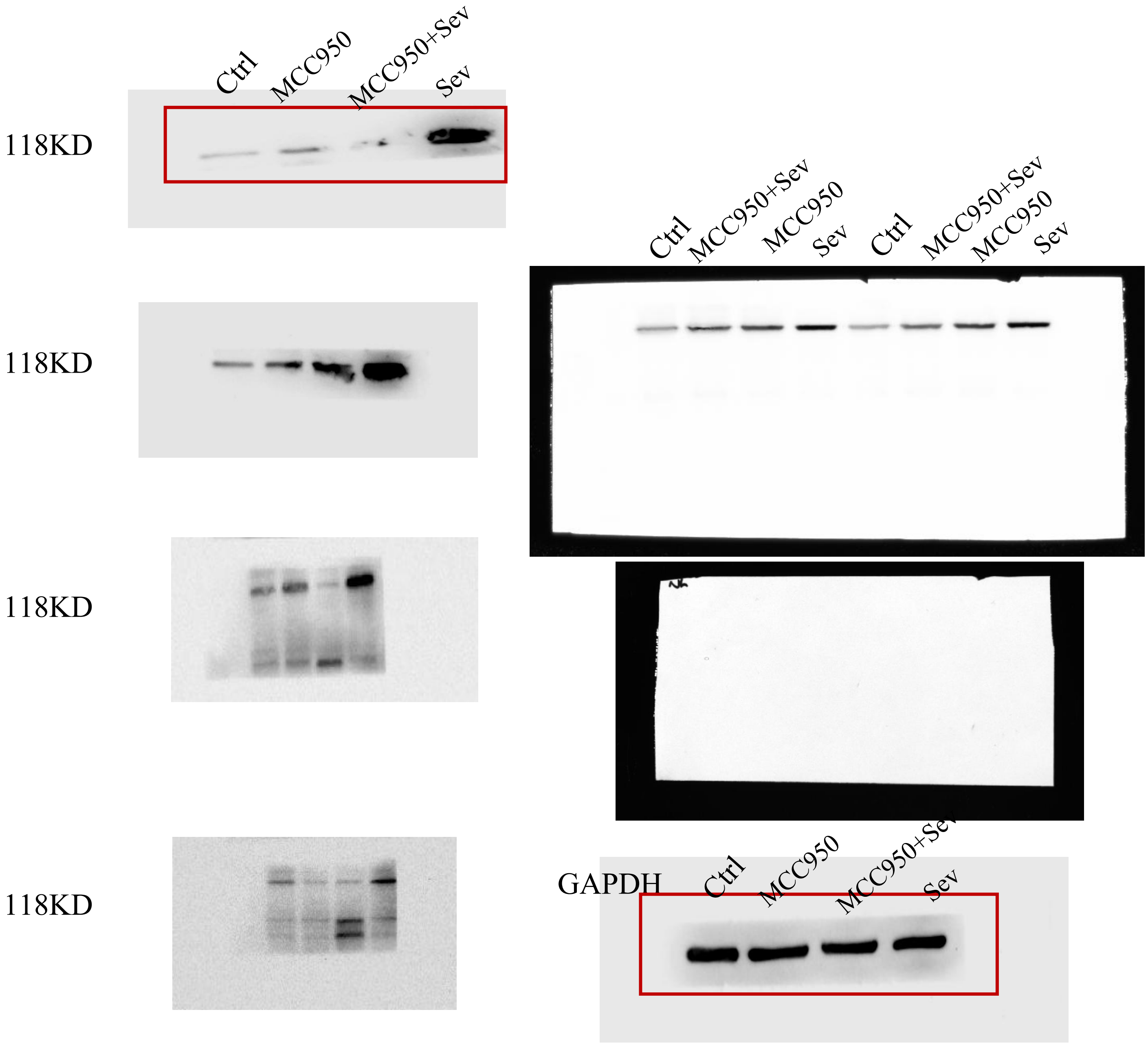

Ctrl
MCC950
MCC950+Sev
Sev
118KD
118KD
118KD
118KD
Ctrl
MCC950+Sev
MCC950
Sev
Ctrl
MCC950+Sev
MCC950
Sev
GAPDH
Ctrl
MCC950
MCC950+Sev
Sev

Caspase-1

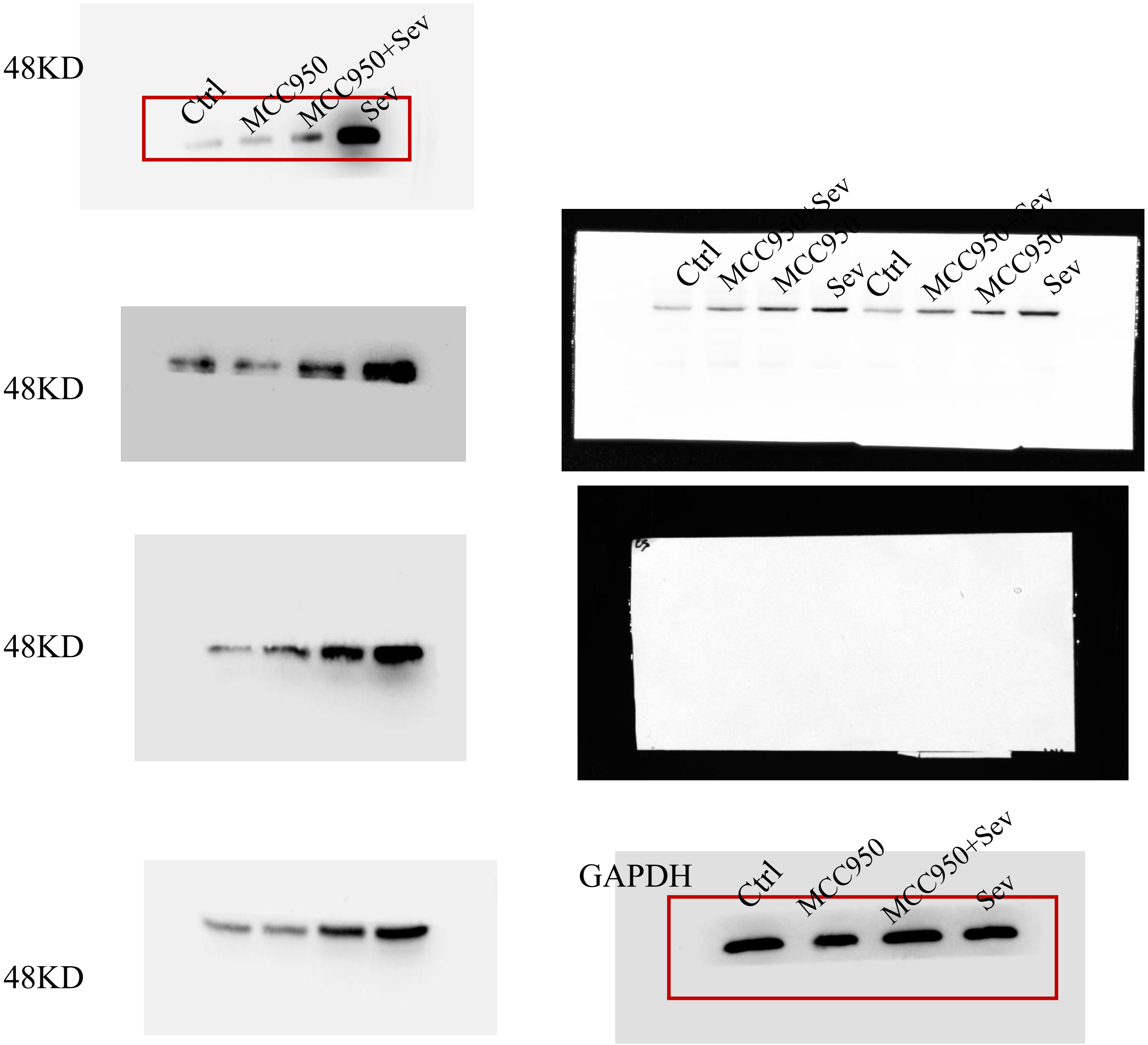
48KD
Ctrl
MCC950
MCC950+Sev
Sev
48KD
48KD
48KD
Ctrl
MCC950+Sev
MCC950
Sev
Ctrl
MCC950+Sev
MCC950
Sev
GAPDH
Ctrl
MCC950
MCC950+Sev
Sev

IL-1β

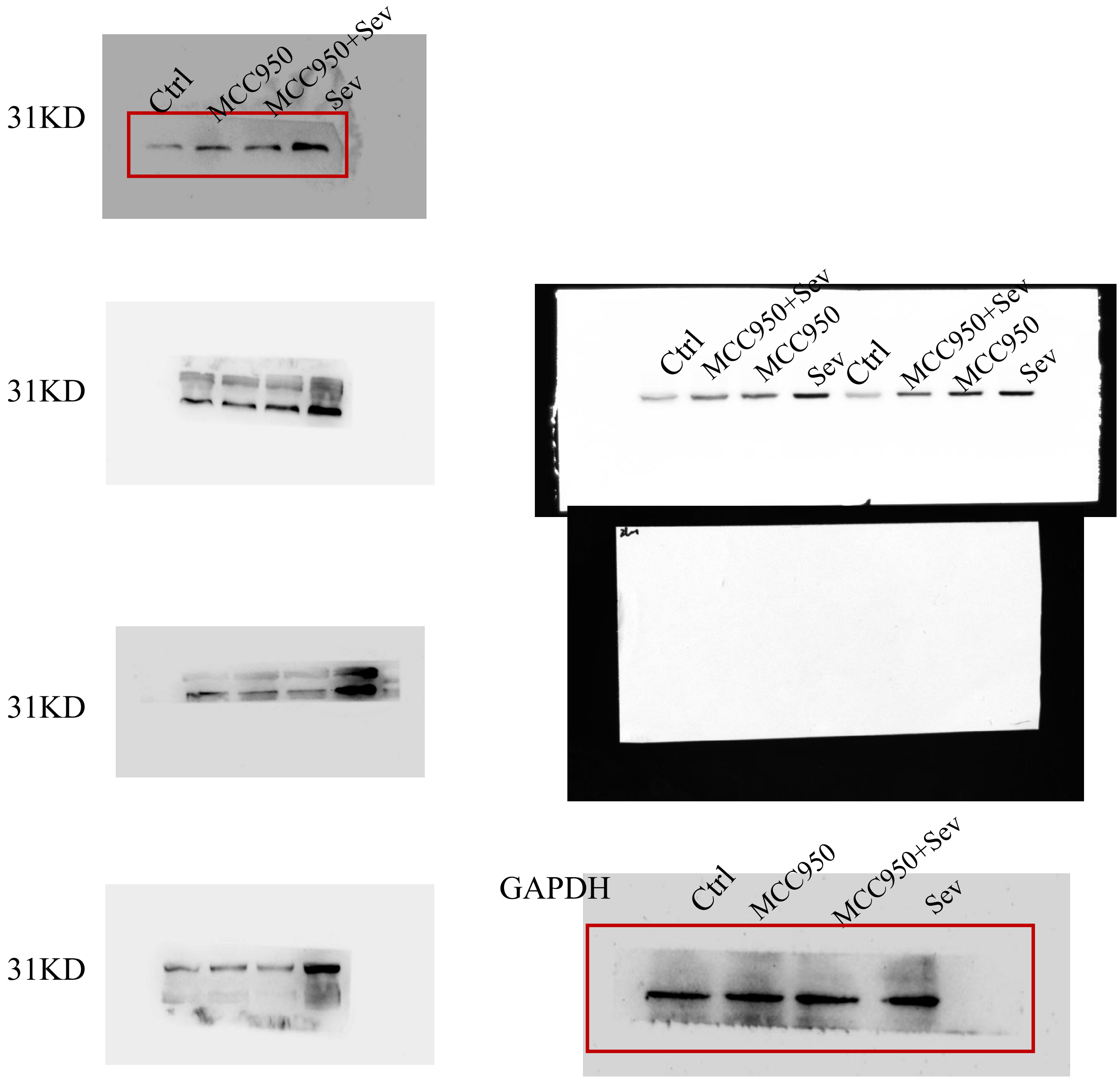

31KD
Ctrl
MCC950
MCC950+Sev
Sev
31KD
31KD
31KD
Ctrl
MCC950+Sev
MCC950
Sev
Ctrl
MCC950+Sev
MCC950
Sev
GAPDH
Ctrl
MCC950
MCC950+Sev
Sev

IL-18

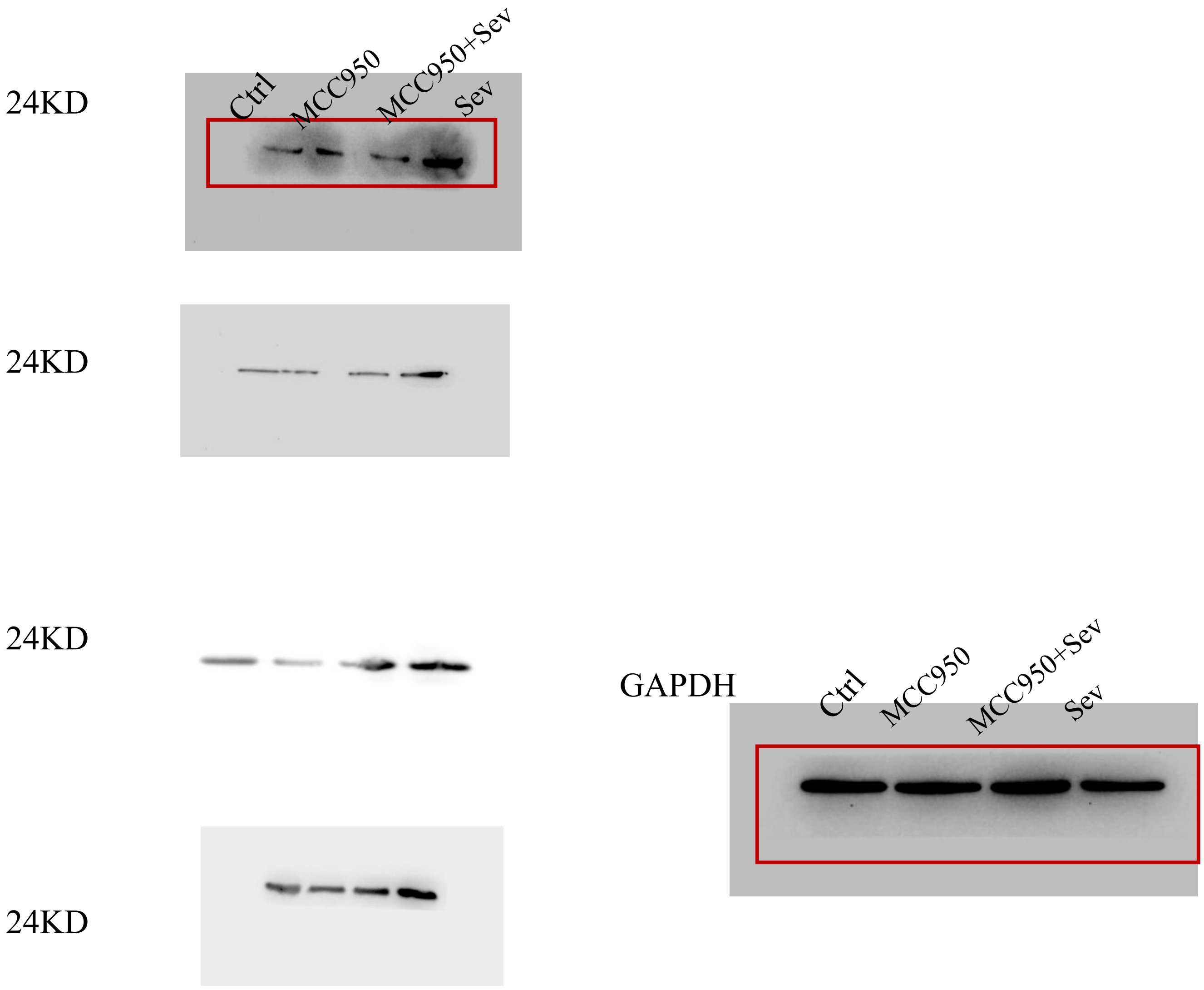

24KD
Ctrl
MCC950
MCC950+Sev
Sev
24KD
24KD
24KD
GAPDH
Ctrl
MCC950
MCC950+Sev
Sev

LC3B

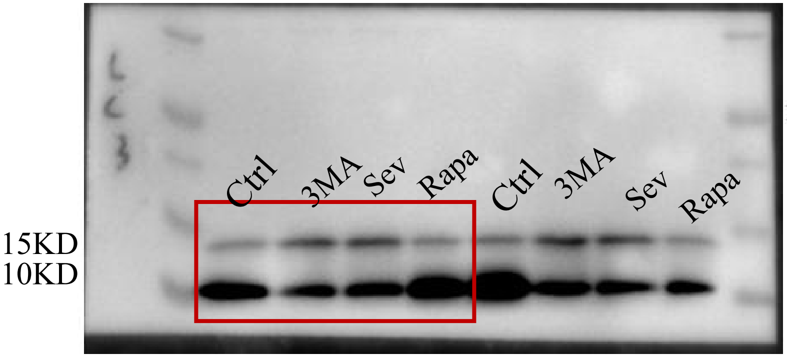
Ctrl
3MA
Sev
Rapa
Ctrl
3MA
Sev
Rapa
15KD
10KD

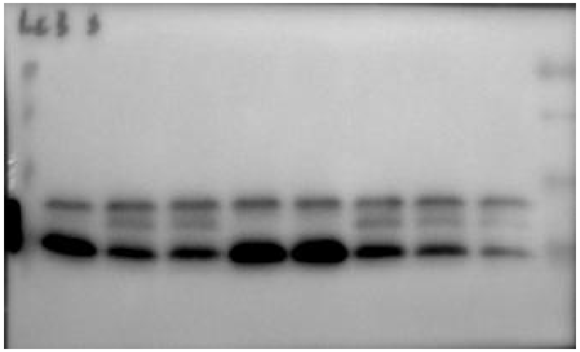
15KD
10KD

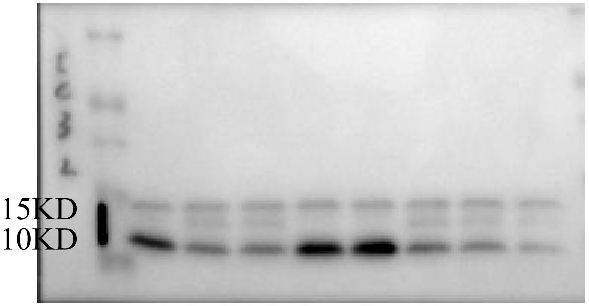
15KD
10KD

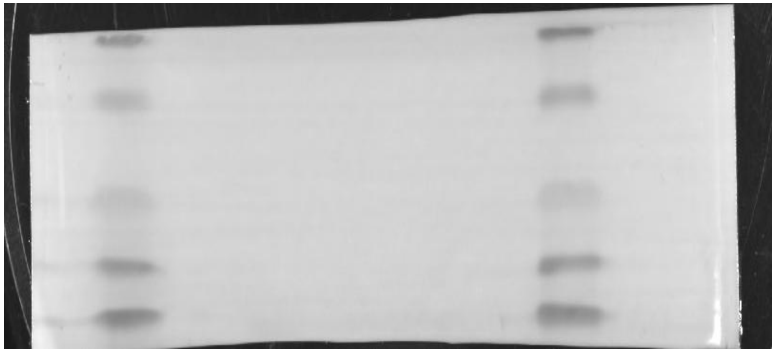

β-actin

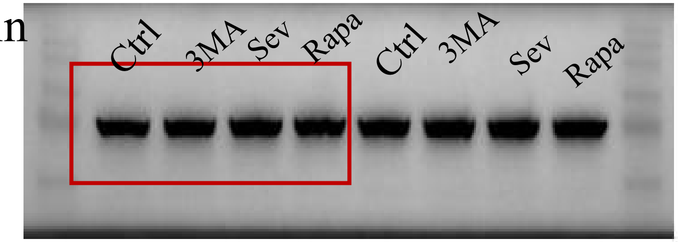
Ctrl
3MA
Sev
Rapa
Ctrl
3MA
Sev
Rapa

P62

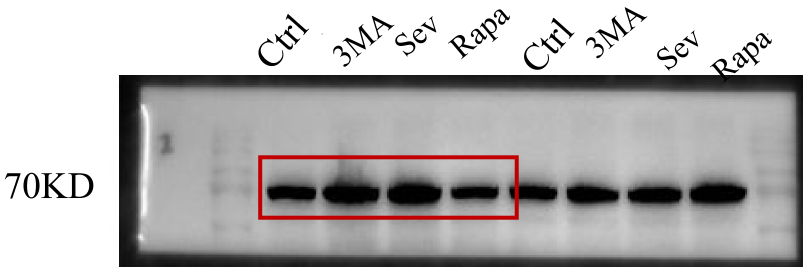
Ctrl
3MA
Sev
Rapa
Ctrl
3MA
Sev
Rapa
70KD

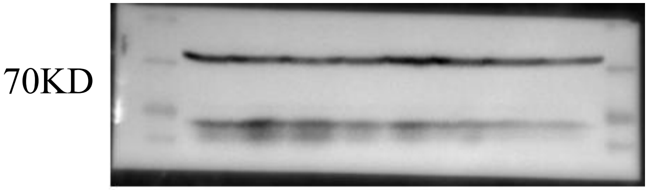
70KD

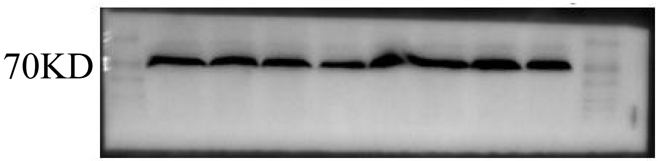
70KD

β-actin

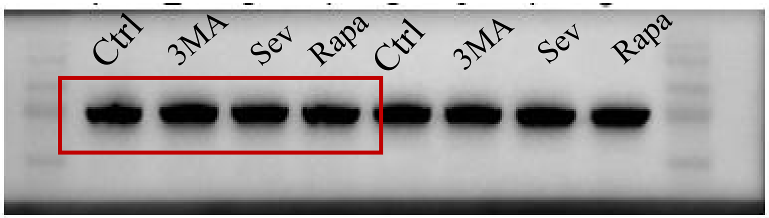
Ctrl
3MA
Sev
Rapa
Ctrl
3MA
Sev
Rapa
